# Supplementary material for: Introducing the Dendrify framework for incorporating dendrites to spiking neural networks
Source: Nat Commun. 2023 Jan 10;14:131. doi: 10.1038/s41467-022-35747-8 (PMC9832130; doi:10.1038/s41467-022-35747-8)
Supplement: Supplementary file 1 — Supplementary Information [file 41467_2022_35747_MOESM1_ESM.pdf]

Introducing the Dendrify framework for incorporating dendrites to spiking neural networks

Supplementary Methods

Simulation accuracy and numerical stability analysis

Biophysically and morphologically detailed models are very stiff systems of equations that require complex implicit numerical methods to solve[4]. However, Dendrify currently depends on Brian's explicit integration methods to solve the equations of the reduced compartmental models. While this approach offers good simulation performance, it also comes with two limitations:

a. The number of a neuron's compartments should be small
b. The simulation's time step (dt) should also be small (not above the 0.05 - 0.1 ms range commonly used)

Nevertheless, with Dendrify, we aim to simply extend the "point-neuron" idea by adding a few compartments that account for specific regions in the dendritic morphology. Thus, our approach typically results in reduced compartmental neuron models that share these characteristics:

1. They have small compartments (usually around 3-5).
2. Each compartment can be quite long (>100 μm).
3. Each compartment is not divided into segments; thus, the number of segments is equal to the number of compartments.

Since Dendrify is commonly used for neuron models with a small number of big compartments, we expect that explicit approaches and a reasonable simulation time step would not cause any substantial numerical issues. To test this hypothesis, we directly compared Dendrify against SpatialNeuron (which utilizes an implicit method) using an adapted version of the 4-compartment model shown in **Fig. 3** and a challenging simulation protocol (see below).

Test details:

- A very high frequency (300 Hz) Poisson input is provided to the most distal dendritic compartment.
- This input generates synaptic currents of fast kinetics (instant rise and 2 ms decay time constant).
- The synaptic weight is large enough to cause robust somatic activation (~8 Hz). Typically, inputs to distal branches of pyramidal neurons fail to do that.

- Simulation time step: Ranged from 0.5 ms or 0.1
- We tested five of Brian's integration methods (Forward Euler, Exponential Euler, 2nd order Runge-Kutta, 4th order Runge-Kutta, and Heun's rule).

Supplementary Figures

```
1  import brian2 as b
2  from brian2.units import *
3  from dendrify import Soma, Dendrite, NeuronModel
4
5  # create soma
6  soma = Soma('soma', model='leakyIF', length=25*um, diameter=25*um)
7
8  # create apical dendrite
9  apical = Dendrite('apical', length=250*um, diameter=2*um)
10
11 # create basal dendrite
12 basal = Dendrite('basal', length=150*um, diameter=2*um)
13
14 # add noise to dendrites
15 apical.noise(tau=20*ms, sigma=3*pA, mean=0*pA)
16 basal.noise(tau=20*ms, sigma=3*pA, mean=0*pA)
17
18 # add synapses
19 apical.synapse('AMPA', pre='cortex', g=1*nS, t_decay=2*ms)
20 apical.synapse('NMDA', pre='cortex', g=1*nS, t_decay=60*ms)
21
22 # merge the compartments into a NeuronModel and set its basic properties
23 edges = [(soma, apical, 10*nS), (soma, basal, 10*nS)]
24 pyr_model = NeuronModel(edges, cm=1*uF/(cm**2), gl=50*uS/(cm**2),
25                         v_rest=-70*mV, r_axial=150*ohm*cm,
26                         scale_factor=3, spine_factor=1.5)
27
28 # create a Brian NeuronGroup and link it to the NeuronModel
29 pyr_group = b.NeuronGroup(4, model=pyr_model.equations, method='euler',
30                         threshold='V_soma > -40*mV', reset='V_soma = -50*mV',
31                         refractory=3*ms, namespace=pyr_model.parameters)
32 pyr_model.link(pyr_group)
```

Supplementary Figure 1 | Python code for the neuron model in Figure 2. *Dendrify* applies a standardized approach for describing the architecture, mechanisms, and parameters of simplified compartmental models. This approach involves creating *Soma/Dendrite* objects (lines 6, 9, 12) representing the model's compartments. Here, soma acts as the primary spiking unit (leaky I&F), while dendrites are simulated (by default) as passive leaky integrators. Users can specify each compartment's physical dimensions, which are used to calculate its surface area. Moreover, *Dendrify* allows adding any desired mechanism (dendritic, synaptic, or other) to a

single compartment, such as Gaussian noise (lines 15, 16) and synaptic currents (lines 19, 20). Users can specify the coupling strength between the adjacent compartments (line 23); otherwise, it is inferred from the model parameters (see Methods). Finally, we introduce another object, the *NeuronModel* (line 24), which has four primary functions: a) to group related *Compartment* objects into a single model, b) to allow setting global model parameters, c) to extract model equations, properties, and custom events, d) to allow deeper integration with *Brian 2,* which unlocks several automations (line 32)*.* Upon creating a *NeuronModel*, users can easily construct a *NeuronGroup* (line 29 - a group of neurons that share the same equations and properties), *Brian's* core object of every simulation. The entire simulation code and detailed *Dendrify* examples are freely available on [*GitHub*](). For more information, see the Methods section and the *Brian 2* documentation: https://brian2.readthedocs.io/en/stable.

```python
1  import brian2 as b
2  from brian2.units import *
3  from dendrify import Soma, Dendrite, NeuronModel
4
5  # create soma
6  soma = Soma('soma', model='leakyIF', length=25*um, diameter=25*um )
7
8  # create trunk
9  trunk = Dendrite('trunk', length=100*um, diameter=2.5*um)
10 trunk.dspikes('Na', threshold=-35*mV, g_rise=34*nS, g_fall=27.2*nS)
11
12 # create proximal dendrite
13 prox = Dendrite('prox', length=100*um, diameter=1*um)
14 prox.synapse('AMPA', pre='pathY', g=0.8*nS,  t_decay=2*ms)
15 prox.synapse('NMDA', pre='pathY', g=0.8*nS,  t_decay=60*ms)
16 prox.dspikes('Na', threshold=-35*mV, g_rise=15.3*nS, g_fall=12.24*nS)
17
18 # create distal dendrite
19 dist = Dendrite('dist', length=100*um, diameter=0.5*um)
20 dist.synapse('AMPA', pre='pathX', g=0.8*nS,  t_decay=2*ms)
21 dist.synapse('NMDA', pre='pathX', g=0.8*nS,  t_decay=60*ms)
22 dist.dspikes('Na', threshold=-35*mV, g_rise=7*nS, g_fall=5.6*nS)
23
24 # merge the compartments into a NeuronModel and set its basic properties
25 edges = [(soma, trunk, 15*nS), (trunk, prox, 10*nS), (prox, dist, 4*nS)]
26 pyr_model = NeuronModel(edges, cm=1*uF/(cm**2), gl=40*uS/(cm**2),
27                         v_rest=-70*mV, r_axial=150*ohm*cm,
28                         scale_factor=2.8, spine_factor=1.5)
29
30 # set dSpike properties
31 pyr_model.dspike_properties('Na', tau_rise=0.6*ms, tau_fall=1.2*ms,
32                             refractory=5*ms, offset_fall=0.2*ms)
33
34 # create a Brian NeuronGroup and link it to the NeuronModel
35 pyr_group = b.NeuronGroup(1, model=pyr_model.equations, method='euler',
36                           threshold='V_soma > -40*mV', reset='V_soma = 40*mV',
37                           refractory=4*ms, events=pyr_model.events,
38                           namespace=pyr_model.parameters)
39 pyr_model.link(pyr_group)
```

Supplementary Figure 2 | Python code for the model shown in Figure 3. The code shown here follows the same principles described in **Supplementary Fig. 1**. In addition, we introduce another feature of *Dendrify,* which is the option to add a dendritic spiking mechanism to *Dendrite* objects (lines 10, 16, 22). Dendritic spiking is modeled in an event-driven fashion, miming the rising and falling phase of dSpikes caused by the sequential activation of inward Na^+ (or Ca^{2+}) and outward K^+ currents (**Fig. 3g**, also see Methods). Users can specify the dSpike threshold and the amplitudes of the inward ('g_rise') and outward ('g_fall') currents individually in each dendrite. Moreover, it is possible to set global dSpike properties (lines 31, 32), such as the decay time constants for the rise and the fall phases, the temporal delay of the fall phase (offset_fall), and a dSpike refractory period.

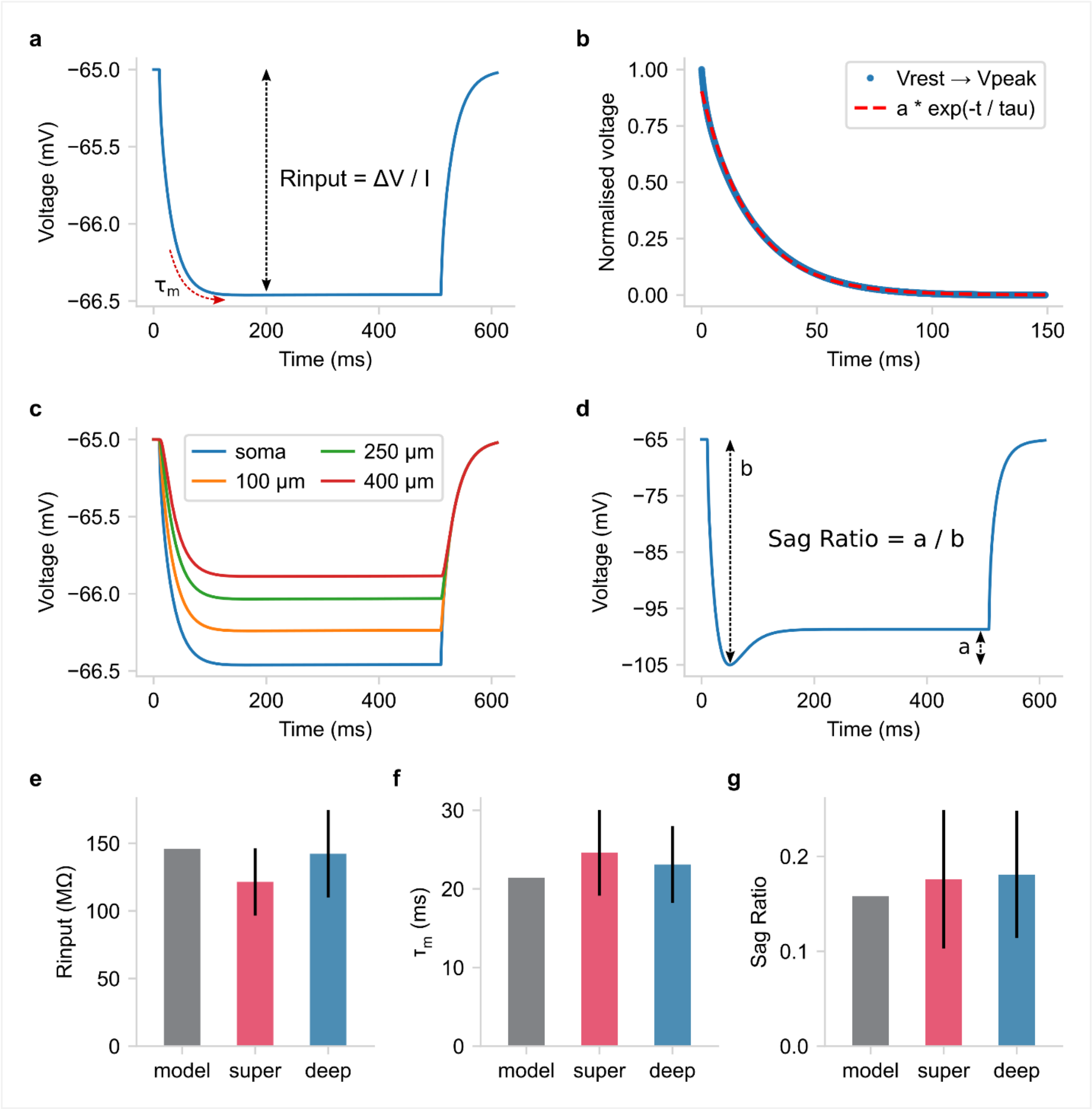

Supplementary Figure 3 | Validation of the passive CA1 PC model properties (relevant to Fig. 4). a-c) Estimating various model properties by replicating an experimental[1], light somatic stimulation protocol (500 ms long somatic current injection of -10 pA amplitude). **a)** Schematic showing the somatic voltage trace used to calculate input resistance (R_{in}). **b)** The membrane time constant (τ_m) was measured by fitting a monoexponential to the somatic membrane hyperpolarization. **c)** Somatic and dendritic voltage traces used to estimate the steady-state, distance-dependent voltage attenuation. **d)** Schematic showing the measurement of the sag ratio by using a strong somatic stimulation protocol[1] to elicit the sag response (500 ms long current injection of -394 pA amplitude to bring the somatic voltage to -105 mV). **e-g)** Comparing model

properties against experimental *in vitro* data[1] regarding deep and superficial PCs of the CA1b Hippocampal region. The experimental values are depicted as means ± std (N_{super} = 29, N_{deep} = 27).

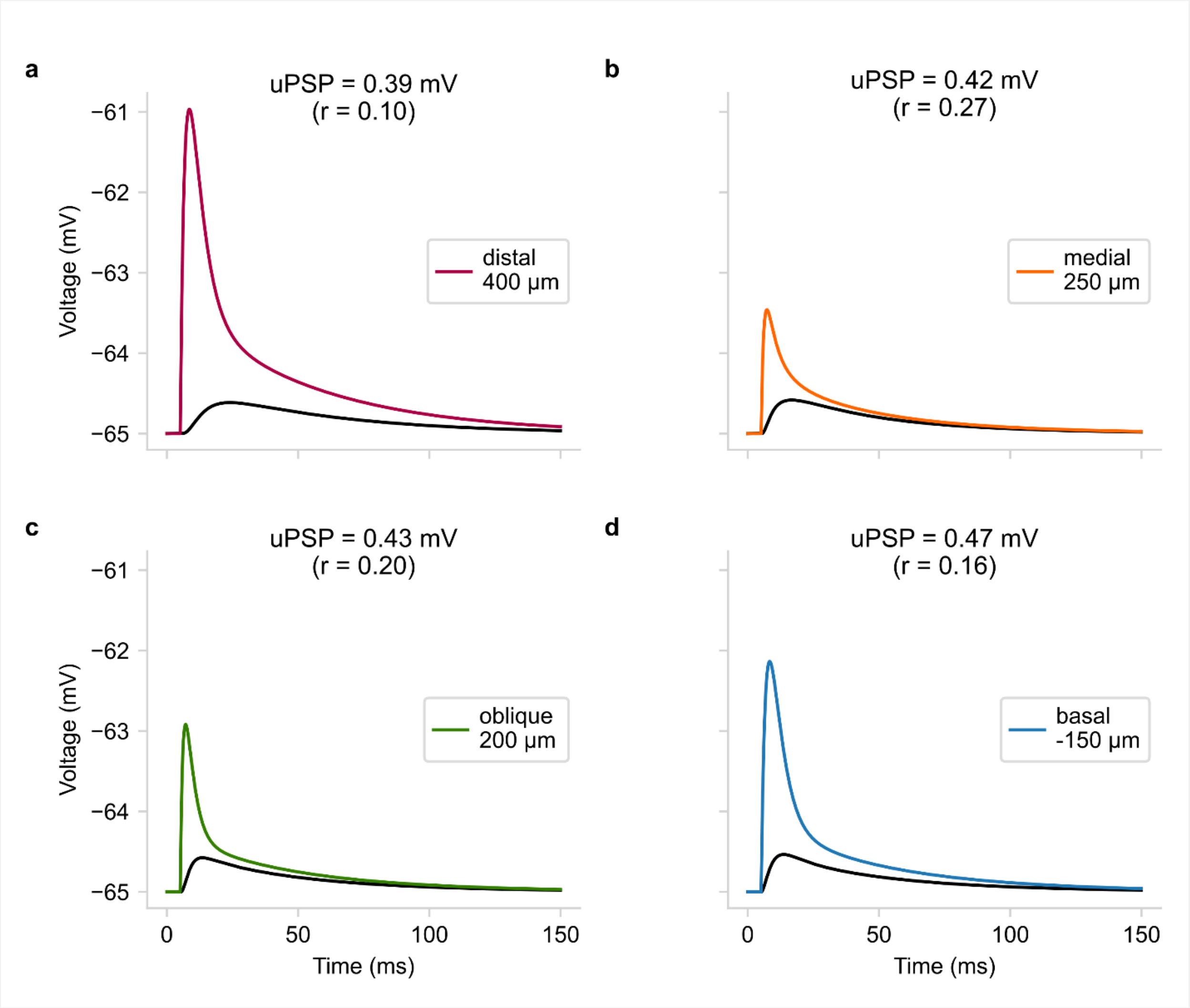

Supplementary Figure 4 | Unitary synaptic responses of the CA1 PC model (relevant to Fig. 4). a-d) Overlay of the dendritic (colored) and the respective somatic (black) voltage responses when a single excitatory synapse (AMPA & NMDA currents) is activated at a distal branch 400 μm from soma (**a**), the medial branch 250 μm from soma (**b**), an oblique branch 200 μm from soma (**c**), a basal branch 150 μm from soma (**d**). Synaptic conductances (g_{AMPA}, g_{NMDA}) were manually adjusted to achieve realistic somatic responses[2]. uPSP: somatic unitary postsynaptic potential. r: the ratio of the somatic to the dendritic peak voltage response ($\Delta V_{soma} / \Delta V_{dend}$).

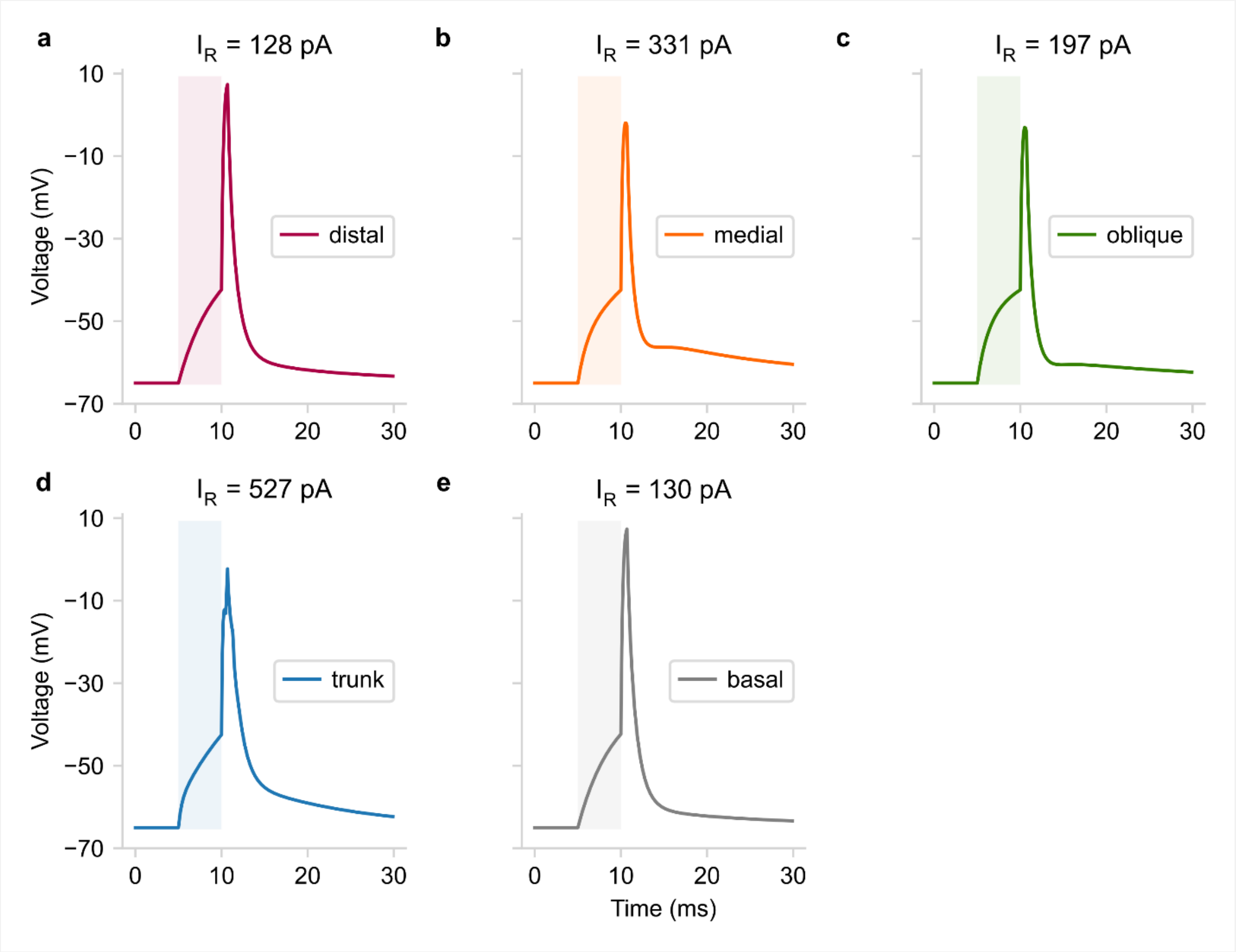

Supplementary Figure 5 | Dendritic spiking in the CA1 PC model (relevant to Fig. 6). a-e) Dendritic voltage responses when constant current of Rheobase amplitude is injected directly into a distal branch (**a**), the medial branch (**b**), an oblique branch (**c**), the trunk (**d**), and a basal branch (**e**). Notice that larger compartments such as the the trunk (**d**) require significantly more current (I_R) to generate a single dSpike than smaller compartments as the distal branches (**a**). Shaded boxes: show the 5 ms long stimulation period (square current pulse). I_R: Rheobase current for evoking a single dendritic current.

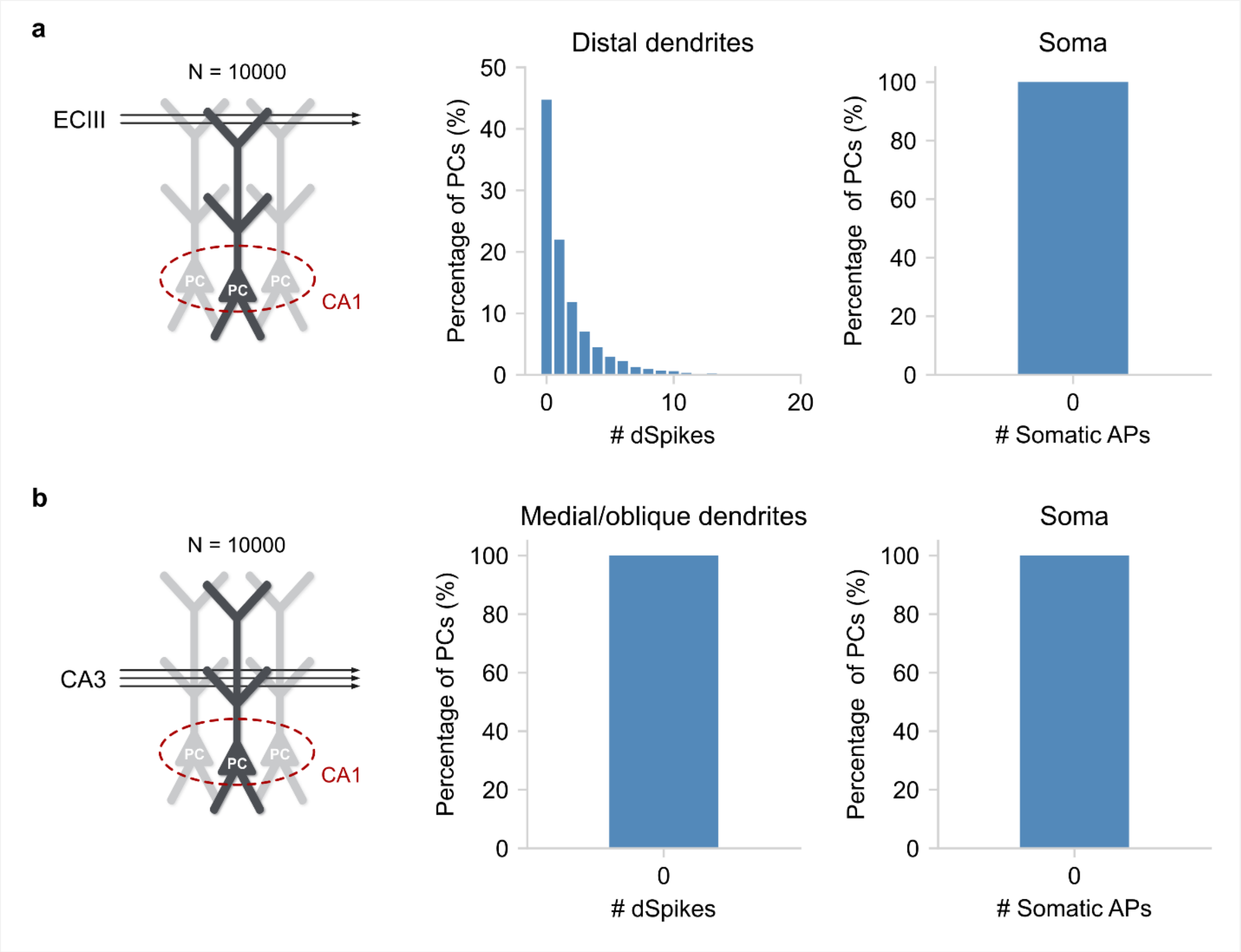

Supplementary Figure 6| Single pathway effect on somatic and dendritic spiking (relevant to Fig. 5). a) When only the ECIII input is administered to a pool of 10,000 PCs, more than half (~55%) will generate at least a single dSpike in one of their distal dendrites. However, due to strong dendritic attenuation, the effect on somatic output is negligible. **b)** When only the CA3 input is available, both the receiver dendrites (medial and oblique branches) and the soma produce subthreshold responses. Notably, both input pathways are simulated as independent Poisson processes, the rate of which is selected to mimic the experiments of Jarsky *et al.* [3].

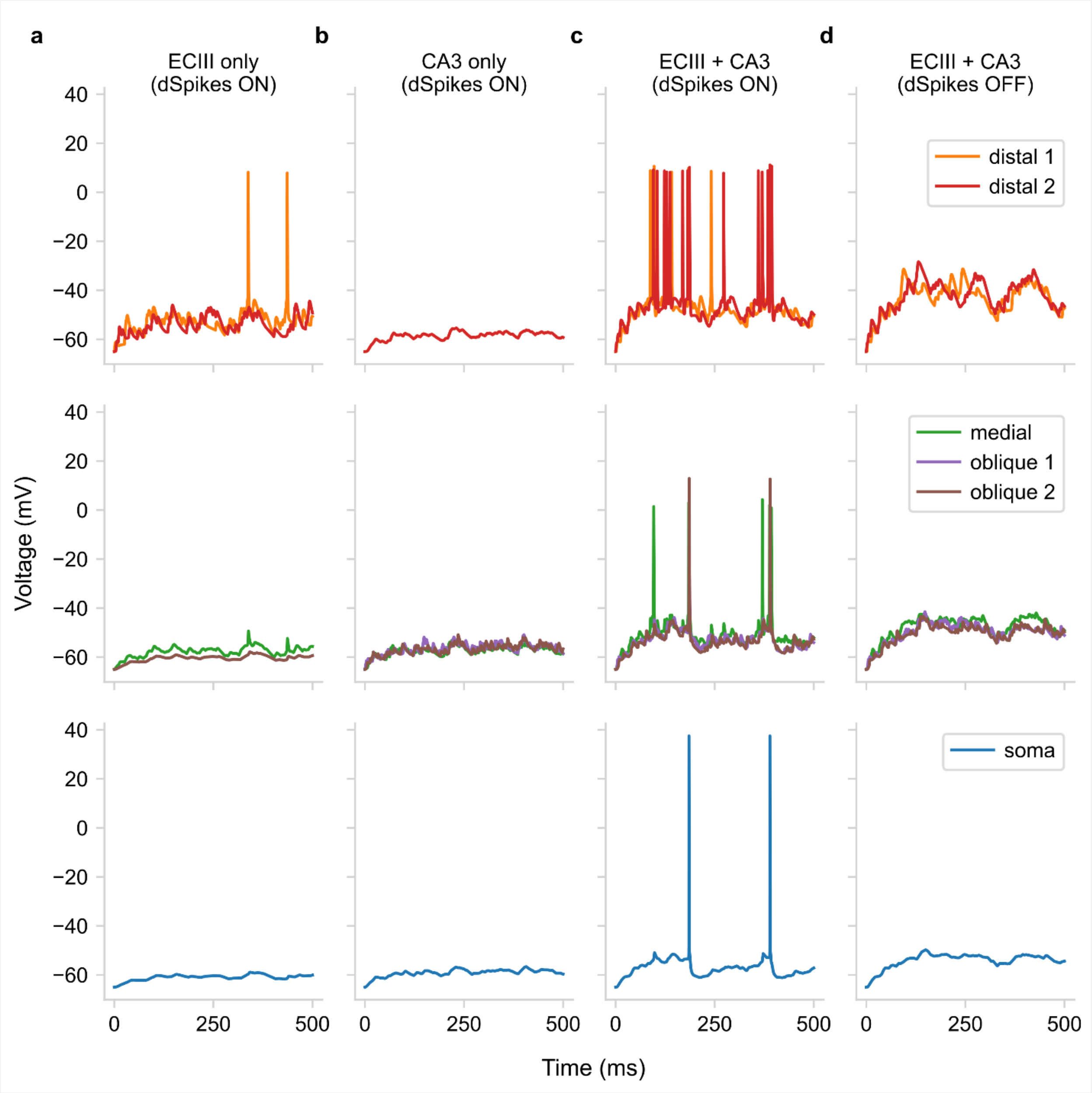

Supplementary Figure 7 | Understanding the role of dendritic Na$^+$ spikes in coincidence detection in CA1 PCs (relevant to Fig. 5). a) When only the ECIII input pathway is active, distal dendrites can generate dSpikes that fail to propagate to the soma due to strong dendritic attenuation. **b)** When only the CA3 input pathway is active, it is not powerful enough to elicit any dendritic or somatic spikes. **c)** When both inputs to the ECIII and CA3 pathways are active, their synergistic effect results in strong dendritic activation that succeeds in activating the soma. **d)** Deactivating dendritic spiking inhibits also deactivates the somatic output even when both inputs to the ECIII and CA3 pathways are active.

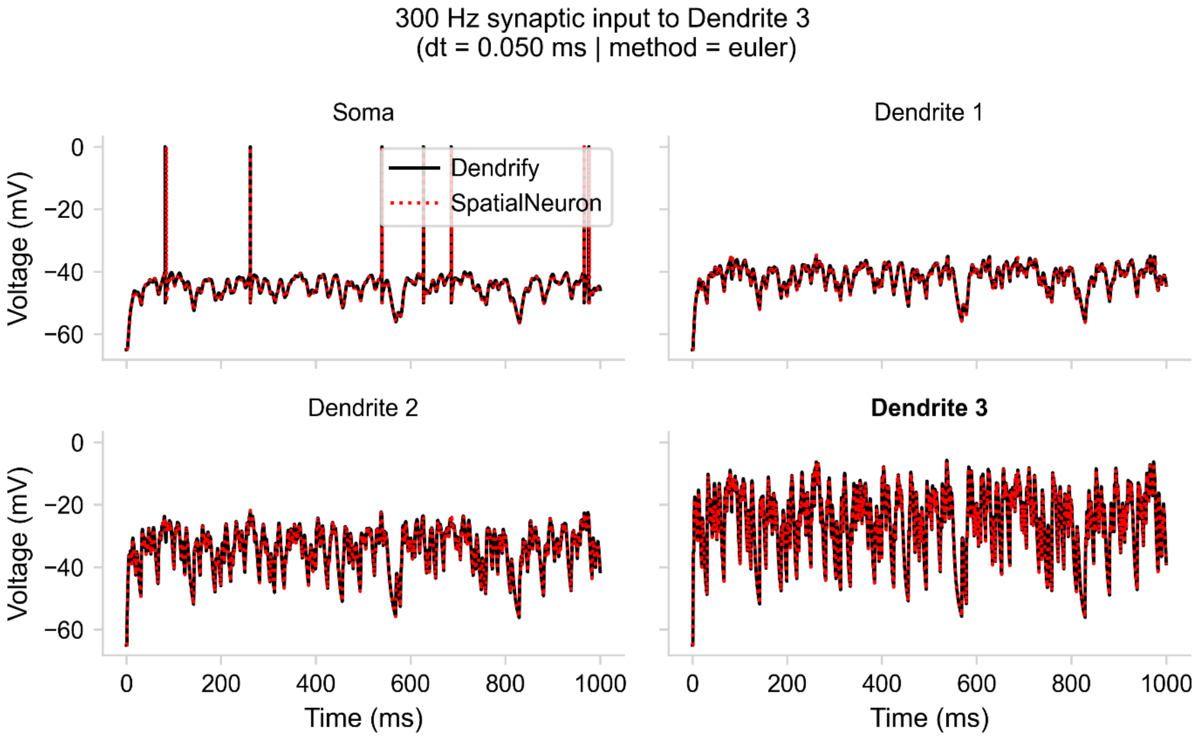

Supplementary Figure 8 | Dendrify vs. SpatialNeuron when using dt = 0.05 ms and the Forward Euler integration method. Voltage responses of all model compartments when high-frequency synaptic input (300 Hz) is provided to the most distal dendrite.

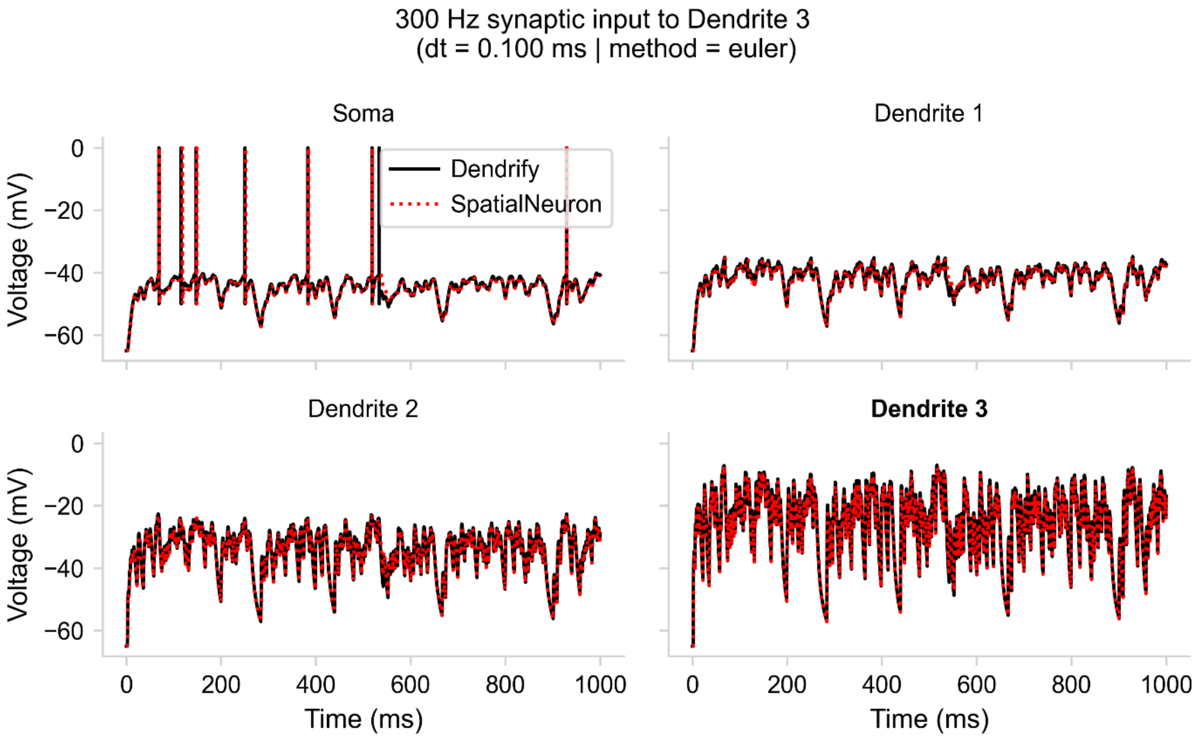

Supplementary Figure 9 | Dendrify vs. SpatialNeuron when using dt = 0.1 ms and the Forward Euler integration method. Voltage responses of all model compartments when high-frequency synaptic input (300 Hz) is provided to the most distal dendrite.

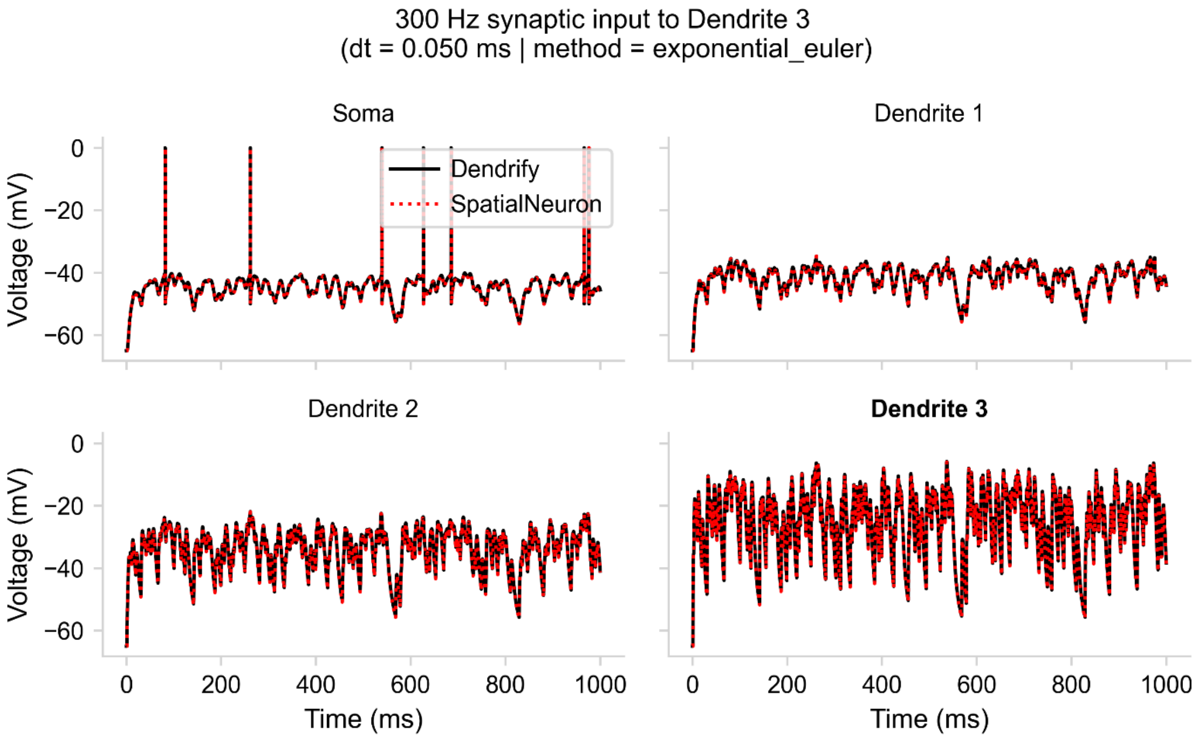

Supplementary Figure 10 | Dendrify vs. SpatialNeuron when using dt = 0.05 ms and the Exponential Euler integration method. Voltage responses of all model compartments when high-frequency synaptic input (300 Hz) is provided to the most distal dendrite.

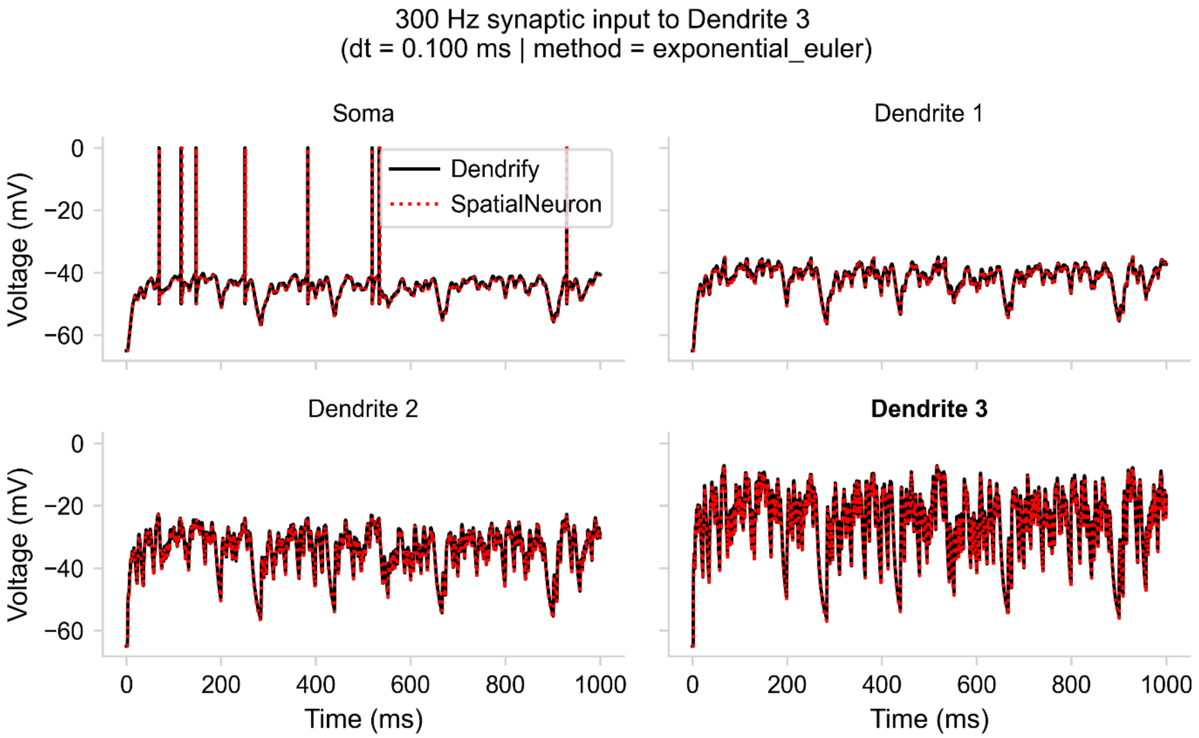

Supplementary Figure 11 | Dendrify vs. SpatialNeuron when using dt = 0.1 ms and the Exponential Euler integration method. Voltage responses of all model compartments when high-frequency synaptic input (300 Hz) is provided to the most distal dendrite.

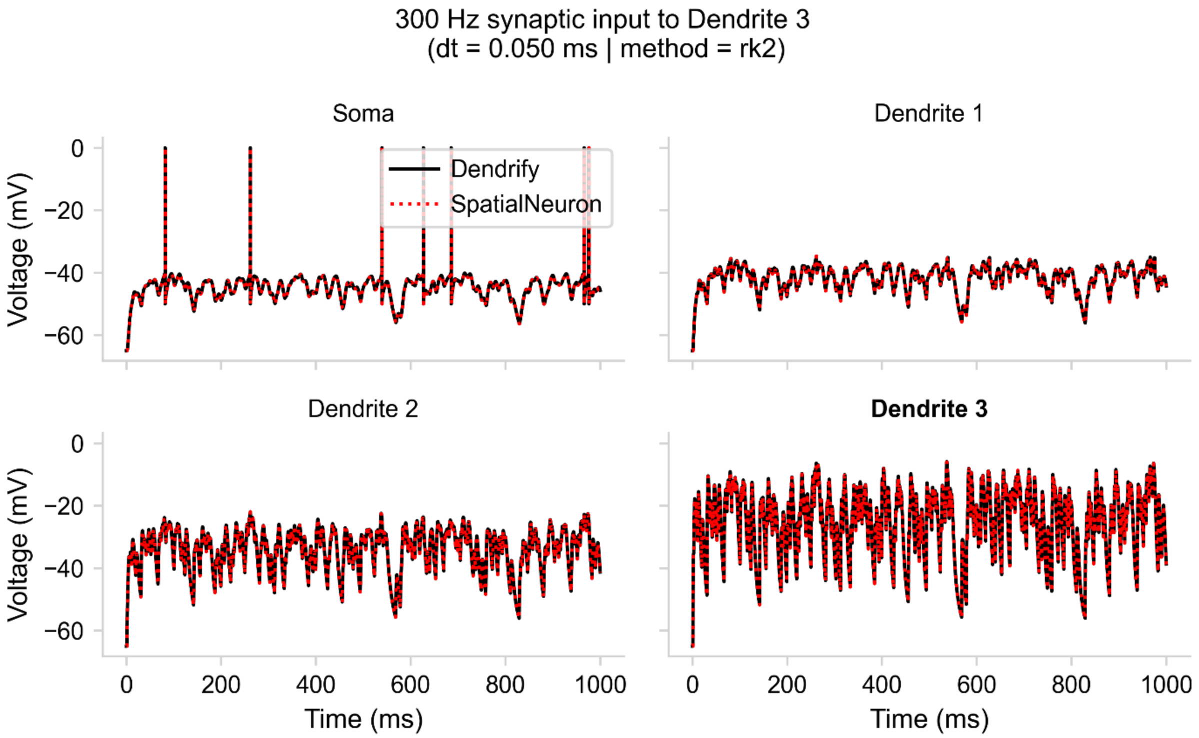

Supplementary Figure 12 | Dendrify vs. SpatialNeuron when using dt = 0.05 ms and a 2nd-order Runge-Kutta integration method. Voltage responses of all model compartments when high-frequency synaptic input (300 Hz) is provided to the most distal dendrite.

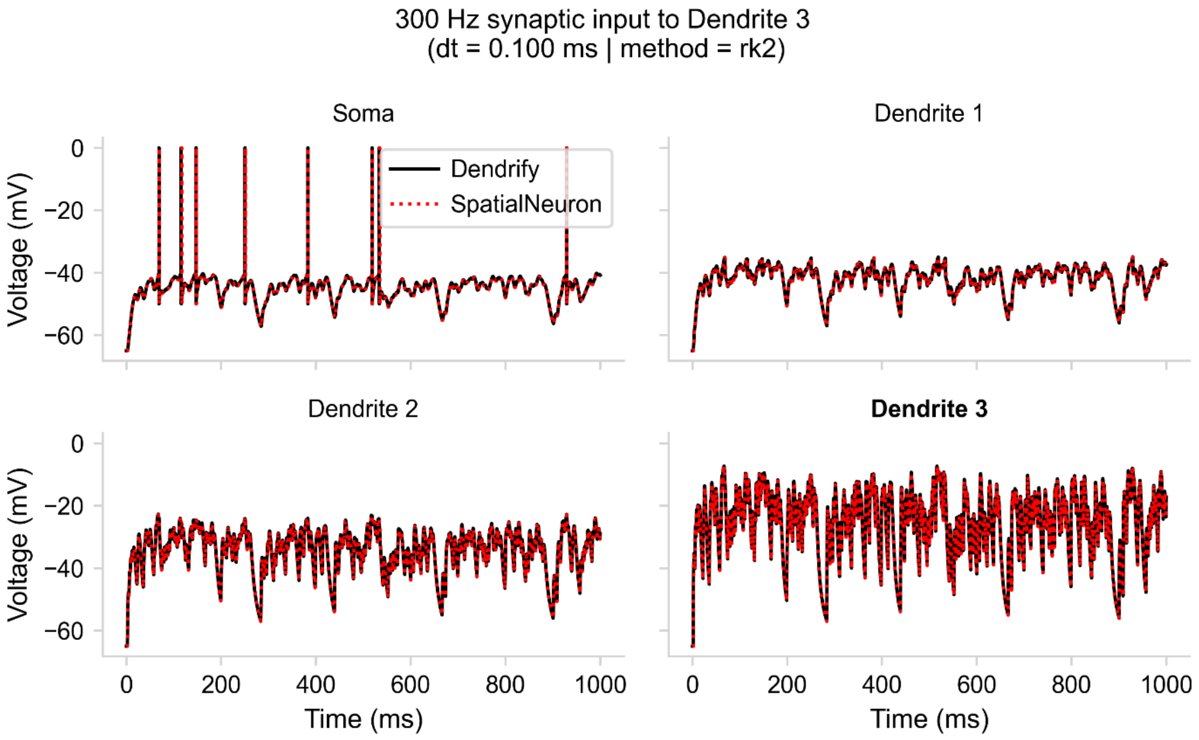

Supplementary Figure 13 | Dendrify vs. SpatialNeuron when using dt = 0.1 ms and a 2nd-order Runge-Kutta integration method. Voltage responses of all model compartments when high-frequency synaptic input (300 Hz) is provided to the most distal dendrite.

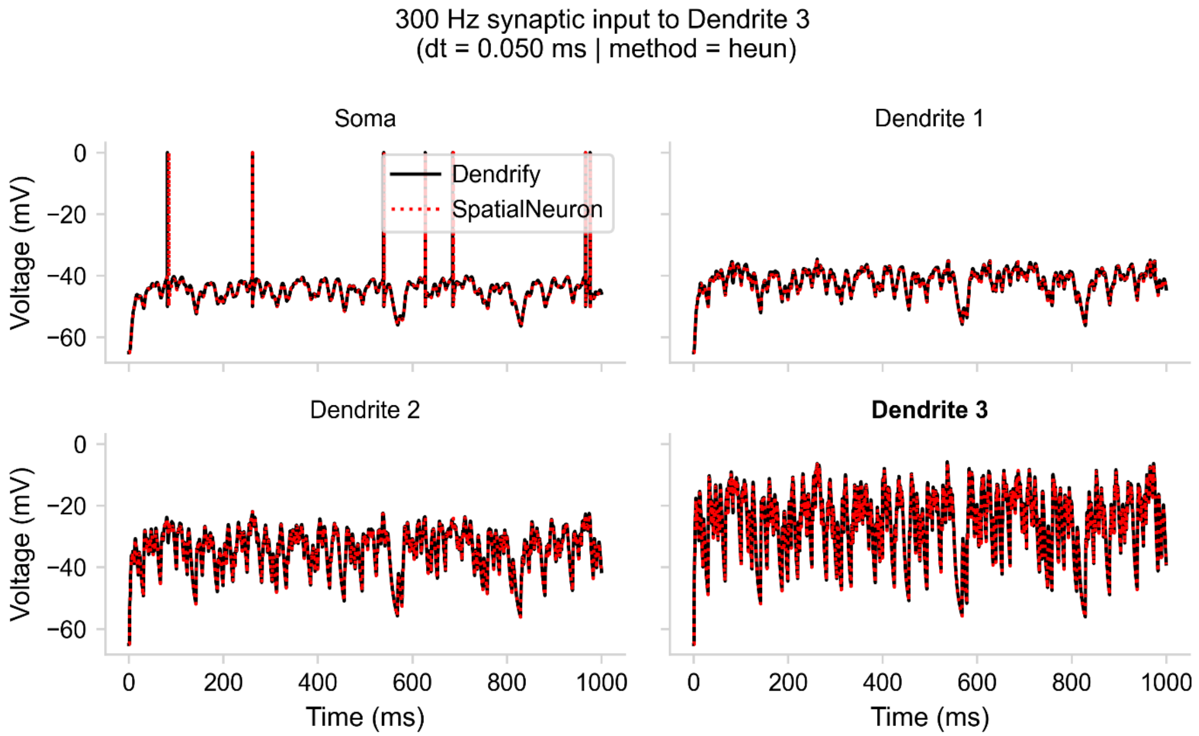

Supplementary Figure 14 | Dendrify vs. SpatialNeuron when using dt = 0.05 ms and Heun's integration method. Voltage responses of all model compartments when high-frequency synaptic input (300 Hz) is provided to the most distal dendrite.

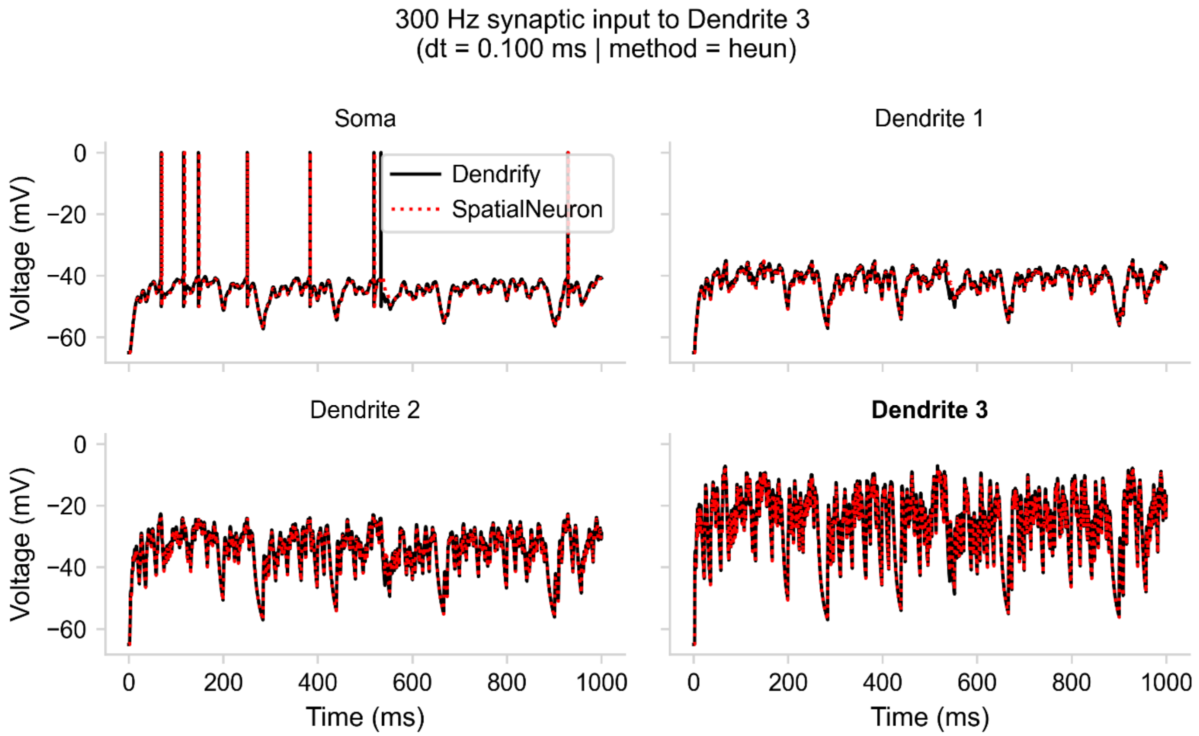

Supplementary Figure 15 | Dendrify vs. SpatialNeuron when using dt = 0.1 ms and Heun's integration method. Voltage responses of all model compartments when high-frequency synaptic input (300 Hz) is provided to the most distal dendrite.

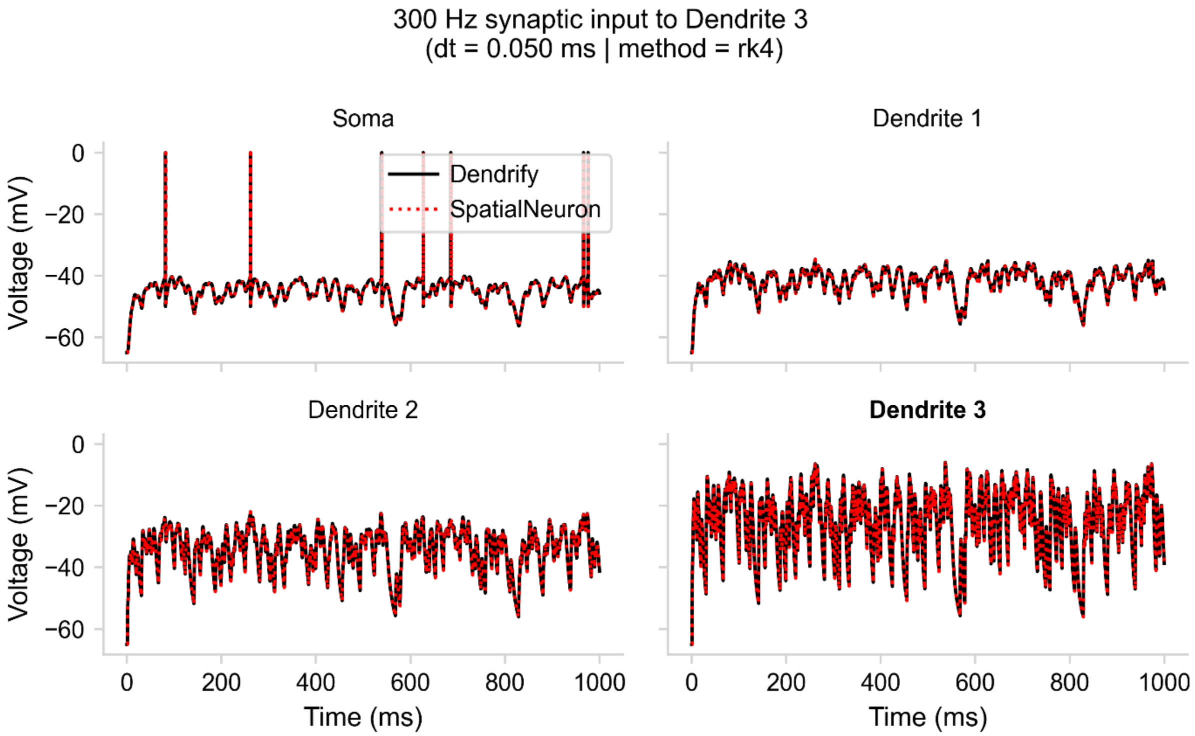

Supplementary Figure 16 | Dendrify vs. SpatialNeuron when using dt = 0.05 ms and a 4th-order Runge-Kutta integration method. Voltage responses of all model compartments when high-frequency synaptic input (300 Hz) is provided to the most distal dendrite.

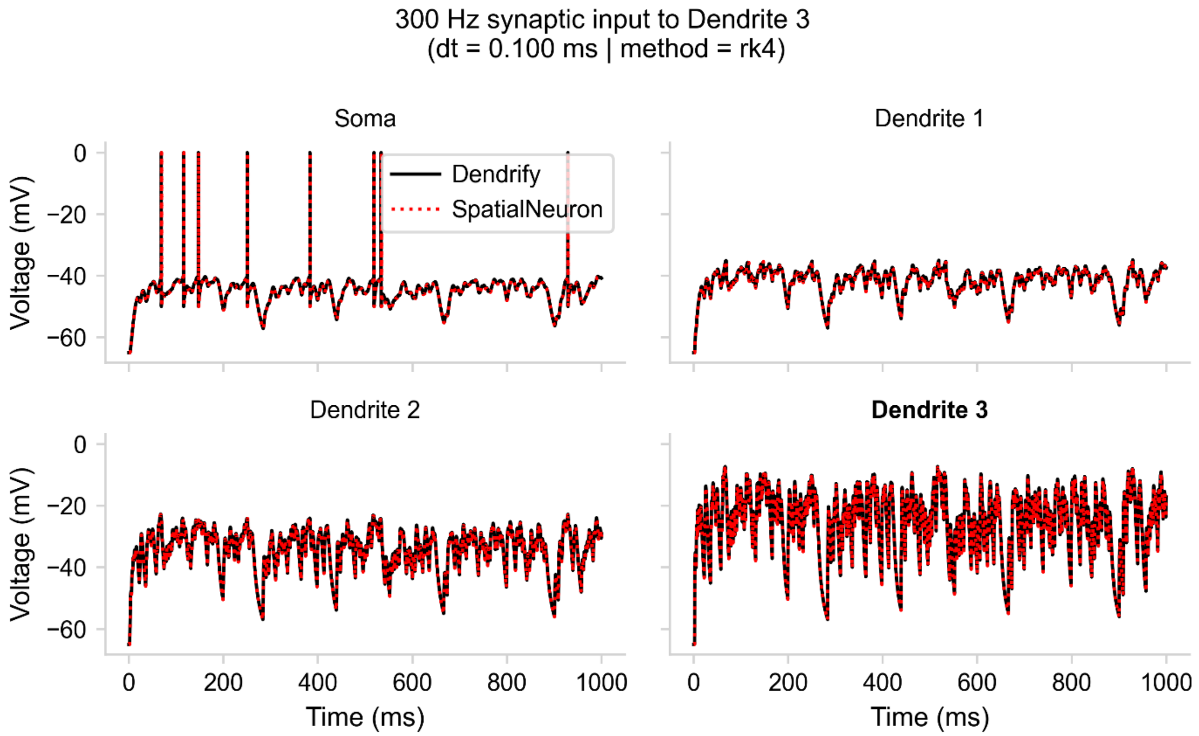

Supplementary Figure 17 | Dendrify vs. SpatialNeuron when using dt = 0.1 ms and a 4th-order Runge-Kutta integration method. Voltage responses of all model compartments when high-frequency synaptic input (300 Hz) is provided to the most distal dendrite.

Supplementary Tables

**Supplementary Table 1 | Parameters for the model shown in Figure 2**		
Timestep	dt	0.1 ms
Specific membrane capacitance	C_m	1 μF · cm^{-2}
Specific leak conductance	g_L	50 μS · cm^{-2}
Axial resistance	r_a	150 Ω · cm
Resting potential (all compartments)	V_{rest}	-70 mV
Spiking threshold	V_{th}	-40 mV
Voltage reset after spike	V_r	-50 mV
Refractory period after spike	t_{ref}	3 ms
Length soma	L_{soma}	25 μm
Diameter soma	D_{soma}	25 μm
Length apical	L_{apical}	250 μm
Diameter apical	D_{apical}	2 μm
Length basal	L_{basal}	150 μm
Diameter basal	D_{basal}	2 μm
Area scale factor	sf_{area}	3
Spine area factor	sf_{spines}	1.5
Coupling conductance (soma-apical)	$g_{soma \leftrightarrow apical}$	10 nS
Coupling conductance (soma-basal)	$g_{soma \leftrightarrow basal}$	10 nS
Noise mean intensity	μ_{noise}	0 pA
Noise standard deviation	σ_{noise}	3 pA
Noise time constant	τ_{noise}	20 ms
AMPA conductance	g_{AMPA}	1 nS
AMPA time constant	τ_{AMPA}	2 ms
NMDA conductance	g_{NMDA}	1 nS
NMDA time constant	τ_{NMDA}	60 ms
alpha (NMDA)	α	0.062 mV^{-1}
beta (NMDA)	β	3.57 mM
gamma (NMDA)	γ	0 mV
AMPA / NMDA reversal potential	E_{AMPA} / E_{NMDA}	0 mV

Supplementary Table 2 | Parameters for the model shown in Figure 3		
Timestep	dt	0.1 ms
Specific membrane capacitance	C_m	1 µF · cm^{-2}
Specific leak conductance	g_L	40 µS · cm^{-2}
Axial resistance	r_a	150 Ω · cm
Resting potential (all compartments)	V_{rest}	-70 mV
Spiking threshold	V_{th}	-40 mV
1st voltage reset after spike	V_{r1}	40 mV
2nd voltage reset after spike	V_{r1}	-55 mV
Spike duration	d_{AP}	0.5 ms
Refractory period after spike	t_{ref}	5 ms
Length soma	L_{soma}	25 µm
Diameter soma	D_{soma}	25 µm
Length proximal	L_{prox}	100 µm
Diameter proximal	D_{prox}	2.5 µm
Length medial	L_{med}	100 µm
Diameter medial	D_{med}	1 µm
Length distal	L_{dist}	100 µm
Diameter distal	D_{dist}	0.5 µm
Area scale factor	sf_{area}	2.8
Spine area factor	sf_{spines}	1.5
Coupling conductance (soma-prox)	$g_{soma\leftrightarrow prox}$	15 nS
Coupling conductance (prox-med)	$g_{prox\leftrightarrow med}$	10 nS
Coupling conductance (med-dist)	$g_{med\leftrightarrow dist}$	4 nS
AMPA conductance	g_{AMPA}	0.8 nS
AMPA time constant	τ_{AMPA}	2 ms
NMDA conductance	g_{NMDA}	0.8 nS
NMDA time constant	τ_{NMDA}	60 ms
alpha (NMDA)	α	0.062 mV^{-1}
beta (NMDA)	β	3.57 mM
gamma (NMDA)	γ	0 mV
AMPA / NMDA reversal potential	E_{AMPA} / E_{NMDA}	0 mV
dSpike rise time constant	τ_{rise}	0.6 ms
dSpike fall time constant	τ_{decay}	1.2 ms
Refractory period after dSpike		5 ms
Offset of dSpike fall		0.2 ms

**Supplementary Table 3 | Parameters for the CA1 PC model shown in Figure 4**		
Timestep	dt	0.1 ms
Specific membrane capacitance	C_m	1 µF · cm^{-2}
Specific leak conductance	g_L	40 µS · cm^{-2}
Axial resistance	r_a	120 Ω · cm
Resting potential (all compartments)	V_{rest}	-65 mV
Spiking threshold	V_{th}	-47.5 mV
Subthreshold adaptation activation voltage	V_a	-65 mV
Time constant of adaptation	τ_a	45 ms
Max subthreshold adaptation conductance	g_a	0.15 nS
Spike-triggered adaptation	Δg_α	21 nS
1st voltage reset after spike	V_{r1}	37.5 mV
2nd voltage reset after spike	V_{r1}	-53 mV
Spike duration	d_{AP}	0.8 ms
Refractory period after spike	t_{ref}	4 ms
Length soma	L_{soma}	30 µm
Diameter soma	D_{soma}	20 µm
Length trunk	L_{trunk}	100 µm
Diameter trunk	D_{trunk}	2 µm
Length medial	L_{med}	150 µm
Diameter medial	D_{med}	1.25 µm
Length distal	L_{dist}	150 µm
Diameter distal	D_{dist}	0.8 µm
Length oblique	L_{obl}	100 µm
Diameter oblique	D_{obl}	1 µm
Length basal	L_{bas}	150 µm
Diameter basal	D_{bas}	0.8 µm
Area scale factor	sf_{area}	2.9
Spine area factor	sf_{spines}	1.5
Coupling conductance (soma-basal)	$g_{soma\leftrightarrow basal}$	3.8 nS
Coupling conductance (prox-trunk)	$g_{prox\leftrightarrow trunk}$	22 nS
Coupling conductance* (trunk-oblique)	$g_{trunk\leftrightarrow obl}$	10.48 nS
Coupling conductance* (trunk-medial)	$g_{trunk\leftrightarrow med}$	10.82 nS
Coupling conductance* (medial-distal)	$g_{med\leftrightarrow dist}$	3.96 nS
AMPA reversal potential	E_{AMPA}	0 mV
AMPA time constant	τ_{AMPA}	2 ms

AMPA conductance distal	g_{AMPA_dist}	0.81 nS
AMPA conductance medial	g_{AMPA_med}	0.81 nS
AMPA conductance oblique	g_{AMPA_ob}	0.6 nS
AMPA conductance basal	g_{AMPA_bas}	0.6 nS
NMDA reversal potential	E_{NMDA}	0.35 mV
NMDA time constant	τ_{NMDA}	60 ms
NMDA conductance distal	g_{AMPA_dist}	0.81 nS
NMDA conductance medial	g_{AMPA_med}	0.4 nS
NMDA conductance oblique	g_{AMPA_ob}	0.4 nS
NMDA conductance basal	g_{AMPA_bas}	0.4 nS
Magnesium concentration	$[Mg^{2+}]_o$	1
alpha (NMDA)	α	0.087 mV^{-1}
beta (NMDA)	β	3.57 mM
gamma (NMDA)	γ	10 mV
Sodium reversal potential	E_{Na}	50 mV
Potassium reversal potential	E_K	-90 mV
dSpike threshold		-42.5 mV
dSpike rise time constant		0.5 ms
dSpike fall time constant		1.2 ms
Refractory period after dSpike		4.2 ms
Offset of dSpike fall		0.6 ms
Sodium channels conductance	g_{Na}	10 $mS \cdot cm^{-2}$
Potassium channels conductance	g_K	4 $mS \cdot cm^{-2}$

*Value generated by *Dendrify*

Supplementary Table 4 | Details for the benchmark test shown in Figure 6	
Neuron model	4-compartment model adapted from **Fig. 3**
External input	2 Poisson generators per neuron (50 Hz each)
Synapses	AMPA (instant rise, τ_{decay} = 5 ms)
Simulated time	1 second
Timestep (dt)	0.1 ms
Integration method	Forward Euler
Operating system	Ubuntu 22.04.1
Brian version	2.5.1
Dendrify version	1.0.5
CPU	i7-9750H
RAM	16 GB
Software	Jupyter notebook (%%timeit)
Brian mode	NumPy
Test	Combined build + runtime (mean of 10 runs)
iPad specs	2022 iPad Air with M1 processor & 8 GB of RAM
iPad software	iPad OS 16.1 & the Carnets app

Supplementary References

1. Masurkar, A. V. *et al.* Postsynaptic integrative properties of dorsal CA1 pyramidal neuron subpopulations. *J. Neurophysiol.* (2020) doi:10.1152/JN.00397.2019.

2. Golding, N. L., Mickus, T. J., Katz, Y., Kath, W. L. & Spruston, N. Factors mediating powerful voltage attenuation along CA1 pyramidal neuron dendrites. *J. Physiol.* (2005) doi:10.1113/jphysiol.2005.086793.

3. Jarsky, T., Roxin, A., Kath, W. L. & Spruston, N. Conditional dendritic spike propagation following distal synaptic activation of hippocampal CA1 pyramidal neurons. *Nat. Neurosci.* (2005) doi:10.1038/nn1599.

4. Mascagni, M. V & Sherman, A. S. Numerical Methods for Neuronal Modeling. *Methods* (1989).
